# Inflammatory Pathways in Parkinson's Disease; A BNE Microarray Study

**DOI:** 10.1155/2012/214714

**Published:** 2012-04-02

**Authors:** Pascal. F. Durrenberger, Edna Grünblatt, Francesca S. Fernando, Camelia Maria Monoranu, Jordan Evans, Peter Riederer, Richard Reynolds, David T. Dexter

**Affiliations:** ^1^Centre for Neuroscience, Division of Experimental Medicine, Faculty of Medicine, Imperial College London, Hammersmith Hospital Campus, Du Cane Road, London W12 0NN, UK; ^2^Neurochemistry Laboratory, National Parkinson Foundation Centre of Excellence Research Laboratory, Department of Psychiatry, Psychosomatics and Psychotherapy, University Hospital of Würzburg, D-98070 Würzburg, Germany; ^3^Neurobiochemistry Laboratory, Hospital of Child and Adolescent Psychiatry, University of Zürich, CH-8050 Zürich, Switzerland; ^4^Department of Neuropathology, Institute of Pathology, University of Würzburg, D-97080 Würzburg, Germany

## Abstract

The aetiology of Parkinson's disease (PD) is yet to be fully understood but it is becoming more and more evident that neuronal cell death may be multifactorial in essence. The main focus of PD research is to better understand substantia nigra homeostasis disruption, particularly in relation to the wide-spread deposition of the aberrant protein *α*-synuclein. Microarray technology contributed towards PD research with several studies to date and one gene, *ALDH1A1* (Aldehyde dehydrogenase 1 family, member A1), consistently reappeared across studies including the present study, highlighting dopamine (DA) metabolism dysfunction resulting in oxidative stress and most probably leading to neuronal cell death. Neuronal cell death leads to increased inflammation through the activation of astrocytes and microglia. Using our dataset, we aimed to isolate some of these pathways so to offer potential novel neuroprotective therapeutic avenues. To that effect our study has focused on the upregulation of *P2X7* (purinergic receptor P2X, ligand-gated ion channel, 7) receptor pathway (microglial activation) and on the *NOS3* (nitric oxide synthase 3) pathway (angiogenesis). In summary, although the exact initiator of striatal DA neuronal cell death remains to be determined, based on our analysis, this event does not remain without consequence. Extracellular ATP and reactive astrocytes appear to be responsible for the activation of microglia which in turn release proinflammatory cytokines contributing further to the parkinsonian condition. In addition to tackling oxidative stress pathways we also suggest to reduce microglial and endothelial activation to support neuronal outgrowth.

## 1. Introduction

Parkinson's disease (PD), estimated to affect 1-2% in the population over the age of 65, rises to 3–5% in people over 85 years of age since age is a predisposing factor [1, 2]. Clinically PD symptomology includes both motor and nonmotor manifestations [3]. The cardinal motor symptoms are rigidity, bradykinesia, resting or postural tremor, and postural instability [4]. Nonmotor features include olfactory dysfunction, autonomic dysfunctions, for example, bladder dysfunction, constipation, and neuropsychiatric disturbances, for example, sleep disorders, hallucinations, dementia, and depression [5, 6]. The onset of motor deficits is primarily due to the neurodegeneration of dopaminergic neurones that originate in the substantia nigra pars compacta (SNpc) and terminate in the caudate and putamen. It is estimated that 70% to 80% of striatal DA neurones are lost at the time of first diagnosis of the motor symptoms [7]. Dopaminergic replacement strategies in the form of L-DOPA or dopamine agonists form the principal strategies for treating PD but such therapeutic approaches are often associated with long-term loss of efficacy and development of major side effects [8].

The aetiology of PD has yet to be fully understood but it is becoming more and more evident that neuronal cell death is a result of not just one event and that PD may be multifactorial in essence supporting a “multiple hit” hypothesis of neurodegeneration [9, 10]. Several events leading to neuronal cell death have been isolated; however the sequence of order in which these events occur remains to be determined. A consensus is emerging suggesting that the crossing of a gene-environment susceptibility threshold yet to be defined is responsible for initiating a cascade of several events such as excitotoxicity, oxidative stress, inflammation, protein aggregation, phagocytosis, and mitochondrial dysfunction leading to apoptosis and that these different factors might present a degree of variation in weight dynamics across patients [8, 11].

Microarray gene expression profiling experiments have increased our understanding of molecular pathogenic mechanisms involved in sporadic and familial PD providing new avenues for research. Several microarray studies have been carried out to date and have established transcriptome profiles of the substantia nigra [12–18]. Most studies were whole-tissue based except for one which was conducted exclusively on dopaminergic neurones isolated from postmortem tissue by laser capture [18]. Additionally, most studies have utilised the Affymetrix platform array, except for Bossers et al. (Agilent platform array) [16]. Furthermore, two microarray studies have been conducted on blood samples [19, 20]. Finally, a genomewide meta-analysis of gene sets from the global PD gene expression (GPEX) consortium highlighted novel underexpressed pathways involved in the control of cellular bioenergetics in PD [21]. High throughput whole-genome platforms are data-driven approaches and assume no a priory aetiological hypothesis. We have recently performed a gene expression analysis on RNA extracted from the substantia nigra (SN) dissected from snap frozen tissues from 12 neuropathologically confirmed cases of sporadic PD and from 7 controls with no neurological disorders utilising for the first time the Illumina whole-genome HumanRef8 v2-long-oligonucleotide microarray technology. This study was part of large-scale microarray study of neurodegeneration including several neurodegenerative diseases (manuscript submitted by Durrenberger et al). We had tested several platforms prior adopting the Illumina platform for its advantageous efficiency over 100 genes (unpublished data). Our main aim was to identify potential pathogenic pathways responsible for the neuronal cell loss using microarray technology. A better understanding of neurodegenerative mechanisms could lead to new cellular and molecular targets, which, in turn, may permit the development of more effective and safe therapies. One of the main findings was the detection of numerous significant upregulated genes involved in immune response and inflammatory processes, which will be the focus of this paper.

## 2. Materials and Methods

### 2.1. Tissue Samples

SNpc sample from 12 clinically and neuropathologically diagnosed patients with idiopathic Parkinson's disease and 7 cases with no neurological conditions were obtained from the Parkinson's UK Tissue Bank at Imperial College London and from the Würzburg Brain Bank Centre in Germany. PD cases were chosen for presenting an early-stage disease (Braak stage 4) rather than end stage. Fully informed consent and ethical approval was obtained for the collection and study of postmortem tissue following guidelines recently published by the consortium [22]. Both tissue banks are members of BrainNet Europe Brain Bank Consortium Network (http://www.brainnet-europe.org/). The tissue samples were snap-frozen as small blocks in isopentane on dry ice. Basic details of the cases are provided in Table 1.

### 2.2. Total RNA Extraction

Total RNA was extracted from dissected snap-frozen tissue (<100 mg) using the RNeasy tissue lipid mini kit according to the manufacturer's instructions (Qiagen Ltd, Crawley, UK) and stored at −80°C until further use. RNA concentration and purity was assessed by spectrophotometry (NanoDrop ND1000; NanoDrop Technologies, Delaware, USA). RNA integrity was further assessed using an Agilent 2100 Bioanalyzer and its lab-on-a-chip platform technology (Agilent Technologies UK Ltd, West Lothian, UK). This system integrates several features in addition to the 28S/18S ribosomal ratio to determine a final RNA integrity number (RIN) [23]. Our samples presented an average RIN value of 6.69 ± 0.6. Homogeneity of RNA quality across samples is crucial for effective results of microarray experiments which are entirely dependent on the quality of RNA [24].

### 2.3. Microarray Experiment and Analysis

Gene expression analysis was performed on RNA extracted from snap-frozen tissues with the Illumina whole-genome HumanRef8 v2 BeadChip (Illumina, London, UK). All samples were analysed on the same day under identical conditions. RNA samples were prepared for array analysis using the Illumina TotalPrep-96 RNA Amplification Kit and following the manufacturer's instructions (Ambion/Applied Biosystems, Warrington, UK). First and second strand cDNA was synthesised from 0.5 *μ*g of total RNA and labelled with biotin. The biotin-labelled cRNA were applied to the arrays using the whole-genome gene expression direct hybridisation assay system from Illumina. Finally the BeadChips were scanned using the Illumina BeadArray Reader. The data was extracted using BeadStudio 3.2 (Illumina, London, UK). Data normalisation and gene differential analysis was conducted using the Rosetta error models available in the Rosetta Resolver system (Rosetta Biosoftware, Seattle, WA, USA) [25]. Fold changes and *P* values were generated based on an intensity ratio between control and disease using a conversion pipeline provided by Rosetta. Multiple testing was carried out to eliminate false positives. Intensity values of individual genes will be presented nonconverted. A principal component analysis was first carried out to detect low quality arrays and a cluster analysis (*P* < 0.01) using a hierarchical algorithm (agglomerative) was conducted to detect potential outliers. No low quality arrays or outliers were detected for the PD cohort. Gene lists containing statistically significant (*P* < 0.01) differentially expressed genes were generated. Significant dysregulated genes with fold change superior or equal to 1.5 were given priority. A gene set enrichment analysis (GSEA) was also conducted (enrichment algorithm: Mann-whitney *U*-Test; *P* < 0.05) on all, upregulated and downregulated genes to determine main biological processes and main up- and downregulated biological processes. Pathway Studio software (Ariadne Genomics Inc., Madrid, Spain) was used to assist with biological interpretation.

### 2.4. Quantification of mRNA Expression by RT-qPCR

The two-step real-time reverse transcriptase quantitative polymerase chain reaction (RT-qPCR) was performed using the QuantiTect reverse transcription kit, the QuantiTect SYBR Green kit and with QuantiTect primer assays (Qiagen) as previously described [26]. Briefly, real-time PCR experiments were performed using the Mx3000P real-time PCR system with software version 4.01 (Stratagene, La Jolla, USA). The QuantiTect primer assays are listed in Table 3. For each sample, reactions were set up in duplicate with the following cycling protocol, 95°C for 15 min, 40 cycles with a 3-step program (94°C for 15 s, 55°C for 30 s, and 72°C for 30 s) and a final melting curve analysis with a ramp from 55 to 95°C. Expression levels of target genes were normalised to the levels of the *BECN1* reference gene and calibrated utilising a standard curve method for quantitation. Beclin-1 was found as the most stable gene amongst the most commonly used reference genes [26] and was hence used as our main normaliser. Some results were duplicated using *XPNPEP1*, a novel reference gene determined from our main experimental study of neurodegeneration (paper in preparation).

### 2.5. Statistical Analysis

The following software packages were used GraphPad Prism 5.01 (GraphPad Software Inc, La Jolla, CA, USA) and Microsoft Office Excel 2007 (Microsoft UK Headquarters, Reading, UK). Group difference was established using Rosetta Resolver system for the microarray data and/or a Student *t*-test (2-tailed or 1-tailed whenever appropriate). The Pearson correlation test was used to establish a relationship between 2 variables. Homogeneity of variance was established with the *F* test. Fisher's exact test was used as a nonparametric test to compare gender. Differences were considered statistically significant if the *P* value was <0.05.

## 3. Results

### 3.1. Microarray Data Analysis

Genes of low intensity (<30 signal-intensity-based and with signal *P* > 0.05) were not considered. In addition to the Rosetta error model, a Student *t*-test (2-tailed; 2-sample unequal variance) was conducted on each gene. A total of 1,423 genes remained (808 upregulated and 615 downregulated) with a greater than 1.5 fold change. The full list can be found in Supplemental 1 of the supplementary material available on line at doi: 10.1155/2012/214714. The gene set enrichment analysis (GSEA) determined most representative biological processes for all genes and for up- and downregulated genes. The main upregulated biological processes were related to immune response and angiogenesis, while neuronal related processes were clearly shown to be downregulated as expected (Table 2).

### 3.2. RT-qPCR Confirmation

**Figure 1:**
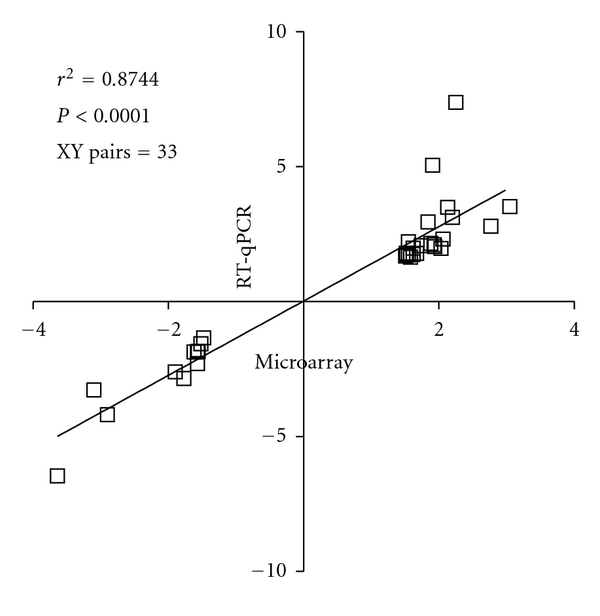
Fold change comparison between the 2 hybridisation techniques. Expression levels from the microarray data of 33 genes were replicated successfully using RT-qPCR. We compared fold changes generated by both hybridisation techniques, conducted a correlation test and found to be a good concordance in expression levels on those 33 genes (*r* ^2^ = 0.8744; *P* < 0.0001).

To confirm some findings from the microarray data, we successfully replicated expression levels from 33 genes using RT-qPCR. Microarray fold changes and *P* values (Rosetta Resolver system and Students *t*-test), qPCR fold changes (expression ratio between the 2 groups), qPCR *P* value (Student *t*-test), and correlation (Pearson) results between microarray and qPCR for each gene are shown in Table 3. Altogether, a good significant correlation between the fold changes from both hybridisation experiments on the 33 genes investigated was found (Figure 1). Only on 2 occasions, expression levels between both experiments did not correlate. Three PD cases showed higher levels of *TNFRSF14* (Tumour necrosis factor receptor superfamily, member 14) with qPCR than with the microarray study. Similarly 2 PD cases showed higher levels of *ELF1* (E74-like factor 1) with qPCR than with the microarray study. Once removed the correlation was significant (data not shown).

### 3.3. Cross-Study Comparative Analysis

**Figure 2:**
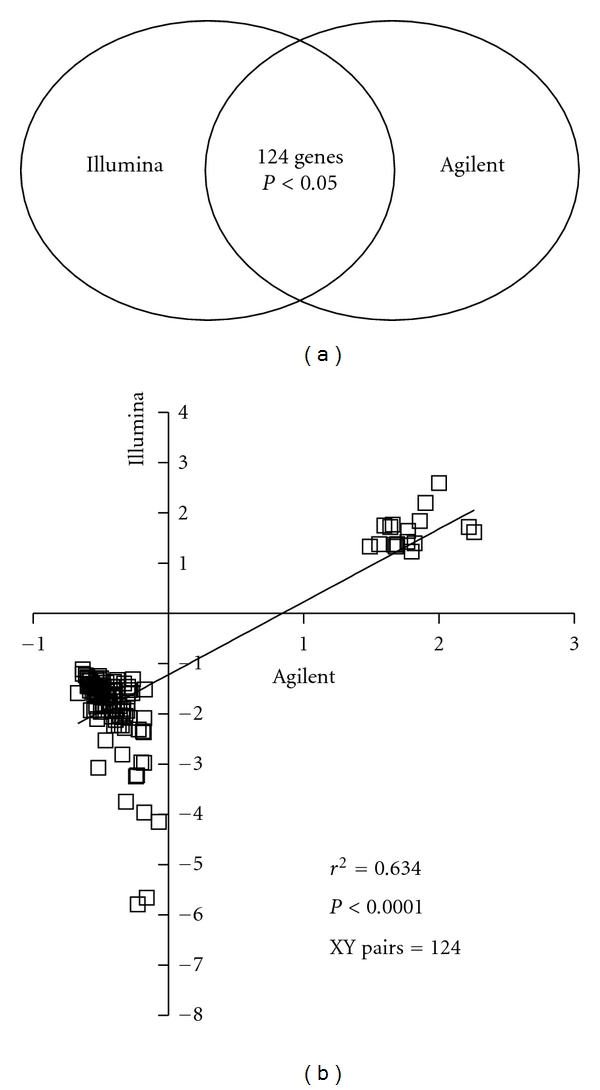
Comparison with Agilent platform. We conducted a direct comparison with the most recent microarray study which was also carried out on a 60-mer oligonucleotide array but using the Agilent platform [16]. 66% of the genes from Bossers et al. (124 genes out of 288 found in represented on both platforms) were also significantly dysregulated in similar fashion as in our dataset (a) and there was a good concordance based on fold changes over the 124 genes (*r* ^2^ = 0.634; *P* < 0.001; XY pairs = 124; b).

To further validate our data we compared our list of other published microarray datasets available in the public domain. Firstly, we cross-referenced our gene list with the most recent microarray study which was also carried out on a 60-mer oligonucleotide array but using the Agilent platform [16]. Altogether, 66% of the genes from Bossers et al. (124 genes out of a total of 288 represented on both platforms) were also significantly dysregulated in similar fashion as in our dataset (Figure 2(a)) and there was a good concordance based on fold changes over the 124 genes (*r* ^2^ = 0.634; *P* < 0.001; XY pairs = 124) with perhaps on occasions higher fold changes reported with the Illumina platform (Figure 2(b)).

**Figure 3:**
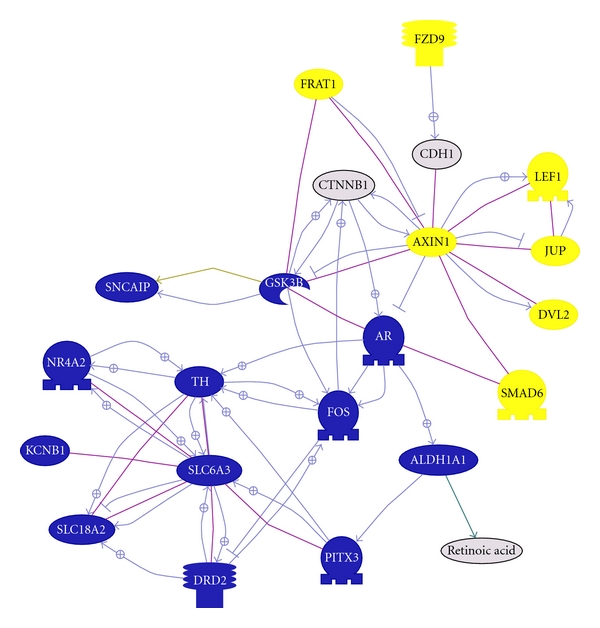
ALDH1A1 pathway. This graph shows all genes from our dataset (except SNCA) known to interact with ALDH1A1. In blue represent genes that are downregulated, in yellow genes that are upregulated and in grey genes not significantly dysregulated but important for the pathway. Line in purple represent binding between two molecules and an arrow with a positive sign represent a positive regulation.

Using an available software tool provided by the Eskitis Institute for Cell and Molecular Therapies, Griffith University (http://ncascr.griffith.edu.au/) containing a reanalysis with differentially expressed gene lists for several studies, we then cross-referenced expression levels of the genes from the Illumina output with 8 gene lists generated from 7 available independent studies at *P* < 0.05 using only the Affymetrix platform [27]. Other microarrays studies have been conducted in PD which were not available within this software tool [21]. Out of 1,423 genes from our list, approximately 40% were significantly dysregulated in at least in one other microarray study. Twelve genes consistently reappeared to be significantly dysregulated across studies (Table 4 and Supplemental 2). *ALDH1A1*, *AGTR1* (angiotensin II receptor, type 1), *ANK1* (ankyrin 1), *ATP8A2* (ATPase, aminophospholipid transporter-like, Class I, type 8A), and *CBLN1* (Cerebellin 1 precursor) were the most consistently reported downregulated gene across studies. These 5 genes suggest deficiencies in oxidation reduction, regulation of vasoconstriction, cytoskeleton organisation (most likely underlying cell proliferation), ATP biosynthetic process, and synaptic transmission (resp.). *ALDH1A1* was consistently (7/8 lists) reported downregulated across microarray studies including ours, that is, various platforms, highlighting dysfunction in DA metabolism but comments on explaining its exact implication in the parkinsonian brain remained inconclusive. Using a pathway analysis software (Pathway studio), we established directly link genes from our dataset with *ALDH1A1* and its pathway will be discussed thereafter (Figure 3).

### 3.4. Inflammatory Pathway

**Figure 4:**
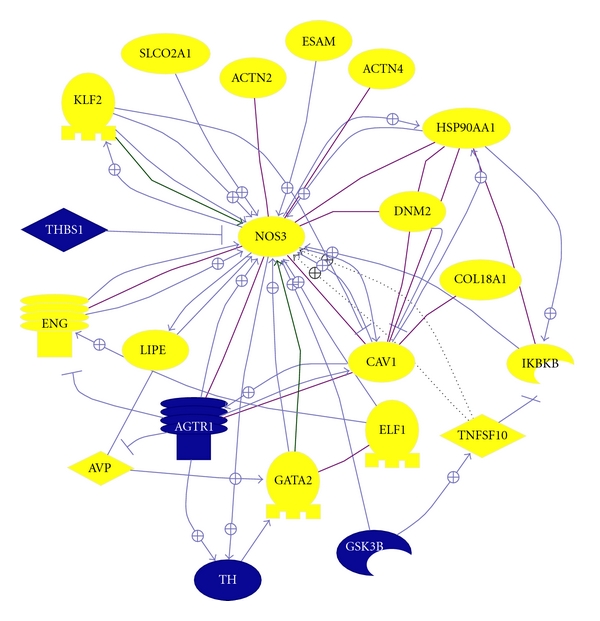
NOS3 pathway. This shows all genes from our dataset (except SNCA) known to interact with ALDH1A1. In blue represent genes that are downregulated and in yellow genes that are upregulated. Line in purple represent binding between two molecules and an arrow with a positive sign represent a positive regulation.

A very strong significant immune-related component was detected by our dataset with the upregulation of numerous immune-related genes covering several categories such as immune or inflammation response, antigen or leukotriene processing, cell proliferation or expansion, and cell adhesion just to mention a few (Table 2). More than 40 genes were found from our list belonging to the category “immune and inflammatory response” just alone. To those we can add several others involved in cell adhesion or proliferation such as integrins. Furthermore, inflammatory response goes hand in hand with angiogenesis since angiogenesis is triggered by hypoxia and inflammation [28]. Two main pathways will be discussed, *P2X7* receptor (P2RX7) and nitric oxide synthase 3 (*NOS3*). Using Pathways Studio, we established directly interacting genes from our dataset with *NOS3* (Figure 4). We have selected to comment on only a selection, mostly on effector molecules or receptors, as they might have potential direct immunomodulatory therapeutic value. *P2RX7* and *NOS3* increases have both been demonstrated by both expression-profiling techniques (Table 3). In addition two other immune-related genes, *TNFRSF14* and *CBLN1*, will be discussed in relation to nervous system development.

## 4. Discussion

One of our main finding was that immune-related genes were strongly significantly upregulated and were predominant compared to genes of other functions. Two pathways were isolated and will be discussed; *P2RX7* and *NOS3* and two additional genes will be commented on: *TNFSFR14* and *CBLN1*. Several microarray studies on parkinsonian substantia nigra have been conducted over the last 7 years using mainly the affymetrix platform. The present study was part of large-scale analysis of genomewide changes in neurodegenerative diseases. The Illumina platform was selected over others for being quality and cost-effective. We placed our gene list in context with some other published gene lists from several independent microarray studies on the substantia nigra to determine concordance levels as well as to determine the most important pathway in the parkinsonian substantia nigra across studies. Our study was the first to our knowledge to use the Illumina platform to investigate abnormal pathways in the parkinsonian brain. Our dataset showed most similarities with the Agilent platform, another 60-mer oligonucleotide array [16] and *ALDH1A1* was most represented across microarray studies [27].

### 4.1. Aldh1a1, Dopamine Metabolism, and Oxidative Stress

Aldehyde dehydrogenase 1 family, member A1 (*ALDH1A1*) was consistently reported downregulated across microarray studies including ours (Table 4); however its exact implication in the parkinsonian brain remains to be fully comprehended. *ALDH1A1* downregulation was mentioned first by Grunblatt et al. [17] and was more comprehensively discussed in Mandel et al. [29]. The Aldehyde dehydrogenase superfamily has recently been extensively reviewed [30]. This cytosolic isoenzyme is ubiquitously expressed in various tissues and in the brain it is highly expressed in dopaminergic neurones of the substantia nigra [31]. It is suggested that Aldh1a1 maintains reduced intraneuronal levels of DOPAL (3,4-dihydroxyphenylacetaldehyde) by catalysing its metabolism to 3,4-dihydroxyphenylacetic acid (DOPAC). This homeostatic function is crucial since evidence showed that accumulation of DOPAL may be neurotoxic and may result in neuronal cell death [32]. Decreased levels of *ALDH1A1* have not been reported in the peripheral blood initially [20] but in another study *ALDH1A1* was reported as being part of a combination of four genes having potential diagnostic value to detect individuals at risk of developing PD [33].

*ALDH1A1* is under the transcriptional control of paired-like homeodomain 3, *PITX3* [34] which is also significantly downregulated in our dataset (fold change: x-2.13, Figure 3). Loss of Pitx3 has been directly linked to selective loss of neurones in the SNpc [35]. Furthermore, Pitx3 is directly linked to dopamine metabolism since Pitx3 was found to regulate tyrosine hydroxylase [36]. Other genes associated with dopamine metabolism were also found downregulated which have been previously reported. These include tyrosine hydroxylase (TH; x-3.1), nuclear receptor subfamily 4, group A, member 2 (NR4A2 formerly known as Nurr1; x-2.96), dopamine receptor D2 (DRD2; x-2.95), solute carrier family 6 (neurotransmitter transporter, dopamine), member 3 (SLC6A3 aka DAT; x-4.1), and solute carrier family 18 (vesicular monoamine), member 2 (SLC18A2 aka VMAT2; x-5.65).

Decreases in gene expression of specific neuronal related genes could be due to the dramatic loss of neurones observed in the substantia nigra. The loss of dopaminergic neurones is estimated to be around 80% [9]. One study, however, estimated that neuronal loss in PD decreased only by 29%. This study showed that neuromelanin-containing neurones sustained most of the loss (51%) while non-neuromelanin-containing neurones increased surprisingly by 104% in PD levelling the loss of total neurones to 29% [16]. The authors consequently suggested that observed decreases of gene expression above 29% in neuronal associated genes could not be accounted solely to neuronal cell loss. Their assumption however is based on the assumption of a linear relationship between gene product and cell number. Only further *in vitro* and *in vivo* studies will help to better understand the ratio between gene products and cell number including compensatory mechanisms.

### 4.2. P2RX7 Pathway and Microglial Activation

**Figure 5:**
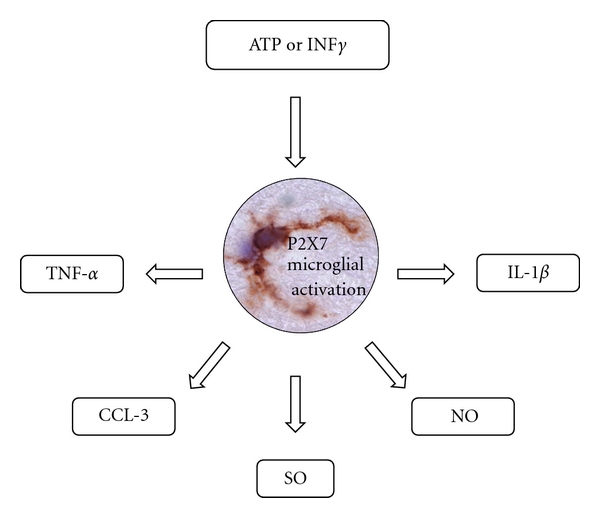
Summary of P2X7 receptor microglial activation. Schematic representation of microglial activation via the P2X7 receptor by extracellular ATP or interferon-*γ* (IFN-*γ*) resulting in the release of tumour necrosis-*α* (TNF-*α*), CC-chemokine ligand 3 (CCL-3), superoxide (SO), nitric oxide (NO), and interleukin-1*β* (IL1-*β*) by microglia. Central picture provided by Durrenberger.

We observed increased *P2RX7* mRNA levels in PD substantia nigra and confirmed expression levels with RT-qPCR. The P2X ionotropic receptors are ATP-gated ion channels and responds to extracellular ATP [37]. ATP is usually released from damaged cells as a result of oxidative stress (as discussed above), ischemia, or inflammation [38]. For instance, astrocytes communicate with Ca^2+^ waves in situation of brain damage or insult with concomitant ATP release and consequently activate microglial purinergic receptors [39]. Purinergic signalling has recently extensively been reviewed [40–42]. Expressed highly on cells of monocyte/macrophage lineage (but also on neurones and astrocytes), once activated, multiple intracellular signalling pathway follows in microglial cells and consequently the release of pro-inflammatory cytokines and chemokines such as tumour necrosis factor-*α* (TNF-*α*) [43], interleukin-1*β* (IL-1*β*) [44], CC-chemokine ligand 3 [45] and the production of superoxide [46], and nitric oxide [47] (summary can be found in Figure 5). Although, studies on postmortem SN tissue and cerebrospinal fluid support elevated levels of inflammatory cytokines such as TNF, IL1*β*, IL-2, IL-4, and IL-6 [48–50], a direct link between microglial activation and PD disease outcome as yet to be determined [51]. However, *in vivo* imaging of microglial activation with the peripheral benzodiazepine receptor binding ligand [11C]-(R) PK11195 in positron emission tomography (PET) scans would suggest an early role of microglia in disease [52]. It is also suggested that early intervention with nonsteroidal anti-inflammatory drugs (NSAIDs) is protective [53]. Furthermore, TNF inhibition has been most successful in rheumatoid arthritis, not so in multiple sclerosis and its effect remains currently to be fully assessed in PD [54]. TNF antagonists may not be effective as a treatment since they will only block one mediator, that is, TNF, hence a more effective approach may be to generally downgrade microglial activation with fluoxetine. *In vivo* and *in vitro* studies have shown fluoxetine to reduce microglial-mediated neurotoxicity and to be a good neuroprotective agent [55].

Furthermore, during an inflammatory response following infection, interferon-*γ* is produced which activates macrophages and increases expression of the *P2RX7* [56]. Interferon gamma was not highly upregulated (fold change: x1.36; *P* = 0.03) but only indications of receptor upregulation activity was revealed from our dataset interferon gamma receptor 1 (*IFNGR1*; x1.55). Upregulation of P2X7 receptor was demonstrated in Alzheimer's disease brain on activated microglia and astrocytes around amyloid plaques [46, 57]. *In vivo* studies demonstrated that amyloid-*β* triggers increases in intracellular Ca^2+^, ATP release, IL-1*β* secretion, and plasma membrane permeabilisation in microglia [58]. The substantia nigra contains the highest concentration of microglia in the brain [59]. P2RX7 were recently investigated in vivo using a rat model of PD and found to be mostly expressed on microglia but also on some astrocytes. Blocking with an antagonist offered partial but significant protection to striatal DA neurones but did not prevent neuronal loss [60]. P2X7 receptor would be a potential candidate for therapeutic intervention since the absence of the receptor has been shown to reduce leukocyte function and the inflammatory response [61]. Even if cell death is not fully prevented it can offer some protection.

### 4.3. NOS3 and Angiogenesis

Angiogenesis has already been highlighted with other microarray studies. *AGTR1* (angiotensin II receptor, type 1), an angiotensin receptor was significantly downregulated in 6/8 microarray studies (Table 4). The neuroprotective effects of blocking the angiotensin receptor 1 on oxidative stress or/and microglial activation have recently been demonstrated in the MPTP model of Parkinson's disease [62] and have been recently reviewed [63]. We would like to focus mainly on endothelial nitric oxide synthase (*eNOS* aka *NOS3*; nitric oxide synthase 3 endothelial cell) which was nearly 2-fold upregulated (x1.91) in PD. Many proteins within our dataset are known to interact with *NOS3* (Figure 4) and some gene products have been validated with RT-PCR. Several proteins are known to directly increase *NOS3* such as heat shock protein 90 (*HSP90AA1*; x1.79), caveolin-1 (*CAV1*; x1.68), *ELF1* (x1.55), endoglin (*ENG*; x1.86), Kruppel-like factor 2 (*KLF2*; x1.6), tumour necrosis factor (ligand) superfamily member 10 (*TNFSF10*; x1.86), dynamin 2 (*DNM2*; x1.69), endothelial cell adhesion molecule (*ESAM*; x2.28), actinin alpha 4 (*ACTN4*; x1.88), thrombospondin 1 (*THBS1* aka TSP1; x-3.08), and GATA binding protein 2 (GATA2; x2.13). Increased levels of eNOS positive cells were found in the SN of a MPTP-mouse model of PD [64]. Moreover, eNOS activity was shown to be reduced when inhibiting SCLCO2A1—a prostaglandin transporter [65]. Endoglin, a transmembrane glycoprotein which plays an important role in vascular integrity and homeostasis, was significantly upregulated (x1.86). In endothelial cells, endoglin is upregulated by hypoxia or TGF-*β* stimulation and downregulated by TNF-*α* [66]. Increased expression of endoglin resulted in eNOS expression [67], while endoglin expression is regulated by *ELF1* [68]. *ELF1* was upregulated in PD substantia nigra (x1.55) and more interestingly was found upregulated (x1.38, *P* = 0.00802) in the peripheral blood of PD patients [20]. This latter evidence shows a direct link between increased expression of a gene product in the peripheral blood and tissue perhaps this gene would prove useful as a potential putative biomarker. Finally, thrombospondin 1 part of the thrombospondin family is an angiogenesis inhibitor and blocks NO-driven angiogenesis. Loss of TSP1 has been associated in animals with increased circulating endothelial precursors, endothelial cell proliferation, and migration [69]. *THBS1* was significantly downregulated (x−3.08) hence resulting in a loss of angiogenesis inhibition. Blocking NO-driven angiogenesis would prove as a potential neuroprotective therapeutic avenue to explore further in the parkinsonian brain even though endothelial dysfunction may not solely be due to disease but also due to levodopa toxicity [70].

### 4.4. Cytokines and Neuronal Development

*TNFRSF14* (aka HVEM, tumour necrosis factor receptor superfamily, member 14 (herpes virus entry mediator)) was significantly upregulated (x1.94) in PD. Expression levels have been replicated (Table 3). Known mostly for facilitating HSV1 entry, this receptor has recently also been shown to play a crucial role in the cell-survival system for lymphoid and epithelial cells [71, 72] and may be responsible for promoting vascular inflammation [73]. Interestingly, increased levels of the cytokine TNFSF14 (aka LIGHT) have been reported in two independent microarray studies conducted on PD blood samples [19, 20]. There are clear signs of genes upregulated in blood and tissue and as with *ELF1* (discussed above), TNFSF14 could also be a potential disease biomarker. Not specific to PD as it was also found upregulated in amyotrophic lateral sclerosis (ALS) and Huntington's disease (HD) perhaps suggesting regional localised activity of this receptor in the cervical spinal cord and basal ganglia (manuscript submitted by Durrenberger et al.). Although TNFSF14 has no known role in the CNS, a very recent study has shown in cultured neurones that LIGHT/HVEM signalling negatively regulated neurite outgrowth from developing sensory neurones [74]. A better understanding of LIGHT/HVEM signalling might prove beneficial for PD and other motor neurones disorders.

Cerebellin 1 precursor (*CBLN1*; x-2.90) was significantly downregulated in 5/8 microarray datasets including the study on dopaminergic neurones (Table 4). Crbln1, belonging to the C1q/tumour necrosis factor subfamily, has only recently been identified as a new transneuronal cytokine (neuromodulator) and was shown to be involved in synapse formation [75]. It was demonstrated that chronic stimulation of neuronal activity by elevating extracellular K(+) levels or by adding kainate to generate kainate-induced seizures decreased rapidly the expression of cbln1 mRNA in mature cerebellar granule cells and that activity-induced reduction was prevented by the addition of exogenous Cbln1 to culture medium [76]. This paradigm could be tested in a PD animal model to determine whether the addition of exogenous Cbln1 could improve neuronal activity in PD.

## 5. Concluding Remarks

In summary, although the initiator of striatal DA neuronal cell death remains to be determined based in our analysis, this event does not remain without consequences. Extracellular ATP, reactive astrocytes appear to be responsible in the activation of microglia which in turn release proinflammatory cytokines contributing further to the parkinsonian condition in the parenchyma. Our data is supportive of a growing body of evidence suggesting that microglial activation plays a key role in the progression of PD with brain rennin-angiotensin systems (RAS) as a key mechanism. In addition to tackling oxidative stress pathways we also suggest to reduce innate immunity activation via the manipulation of the brain's RAS so to enhance and support new neuronal outgrowth. Interactions between these mechanisms as well as perhaps the host's ability to resolve inflammation deserve further investigations

## Supplementary Material

Supplementary Material 1: List of genes significantly *(*P < 0.001) dysregulated and of a fold change above 1.5 *(*Rosetta error model) with gene description, ontology, chip information and raw data on each gene.
Supplemental Material 2: Full list of genes from our dataset that were also significantly dysregulated in another microarray study.

## Figures and Tables

**Table 1:** Basic clinical and neuropathological characteristics of study cases.

Case	Gender	Age at Death (years)	Illness Duration (years)	PM delay (hours)	COD
C01	M	66	N/A	23.00	Global heart failure, hypopharynx carcinoma
C02	M	54	N/A	27.00	Pneumonia, respiratory failure
C03	M	64	N/A	50.00	Kearns-Sayre-Syndrome, resp. failure
C04	M	55	N/A	24.00	Aspiration pneumonia
C05	F	60	N/A	9.00	Circulatory collapse, cutaneous T-cell-lymphoma perianal carcinoma
C06	M	58	N/A	9.00	Unknown
C07	F	104	N/A	9.50	Chest infection

Mean value		64.5 ± 5.85		27.94 ± 4.84	

PD01	F	86	15	5.50	Sudden collapse
PD02	M	78	24	20.25	Unknown
PD03	F	85	18	13.50	Bronchopneumonia, Breast Cancer with metastasis and PD
PD04	F	76	10	13.50	Unknown
PD05	M	77	10	5.50	Unknown
PD06	M	80	19	16.00	Unknown
PD07	M	80	5	7.00	Unknown
PD08	F	80	13	10.00	Old age and PD
PD09	M	86	8	2.50	Ischaemic bowel and atrial fibrillation
PD10	F	87	9	22.00	Gastrointestinal bleeding
PD11	F	81	14	21.50	Unknown
PD12	M	82	11	10.00	Pneumonia, Fractured neck of femur, Pulmonary embolisms, COPD, Dementia

Mean value		81.5 ± 1.07	13 ± 1.54	12.2 ± 1.91	

C: Control; PD: Parkinson's disease; PM: Postmortem; COD: cause of death.

**Table 2:** List of top 20 biological processes for all, up- and downregulated genes (*P* < 0.005).

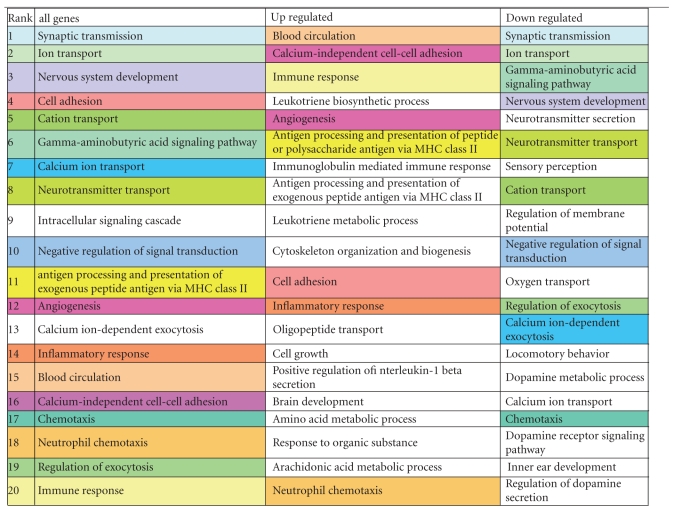

Colours are respectively similar between all genes list to either up- or downregulated genes. Blue/green shades for downregulated; Red/yellow shadesfor upregulated.

**Table 3:** Expression levels of genes validated using qPCR (in alphabetical order).

Name	Description	Function	Primers Assays	Chromosome	Microarray data			RT-qPCR data		Microarray/qPCR	
					FC	*P* value	*t*-test	FC	*P* value	Pearson *r *	*P* value
ACTN4	Actinin, alpha 4	Regulation of actin cytoskeleton	QT00030765	19q13.2	**1.88**	<0.0001	0.0004	**2.155**	0.006	0.8744	0.0002
ASTN1	Astrotactin 1	Neuron adhesion	QT00084609	1q25.2	**−1.52**	<0.0001	0.0006	**−1.56**	0.003	0.6475	0.0067
CBLN1	Cerebellin 1 precursor	Synaptic transmission	QT00224126	16q12.1	**−2.90**	0.0048	0.048	**−4.19**	0.026	0.9745	<0.0001
CLDN11	Claudin 11	Axon ensheathment	QT00008085	3q26.2	**1.62**	0.0048	0.0158	**1.98**	0.014	0.8626	0.0001
CSF1R	Colony stimulating factor 1 receptor	Macrophage function	QT00041293	5q33.1	**1.52**	0.0042	0.035	**1.79**	0.012	0.719	0.0005
CTGF	Connective tissue growth factor	Angiogenesis	QT00052899	6q23.2	**2.77**	0.0001	0.003	**2.79**	0.032	0.9276	<0.0001
DNAH17	Dynein, axonemal, heavy polypeptide 17	Microtubule based movement	QT00065226	17q25.3	**2.06**	0.0007	0.01	**2.32**	0.025	0.8675	0.0001
DOCK10	Dedicator of cytokinesis 10	Unknown	QT00009835	2q36.2	**1.58**	0.0022	0.01	**1.65**	0.014	0.8717	<0.0001
DRD1IP	Dopamine receptor D1 interacting protein	Dopamine receptor signalling	QT00493031	10q26.3	**−1.90**	0.0062	0.037	**−2.6**	0.035	0.9471	<0.0001
ELF1	E74-like factor 1	Immune response	QT00023716	13q14.11	**1.55**	<0.0001	0.0076	**2.21**	0.044	ns	ns
GABRB1	Gamma-aminobutyric acid (GABA) A receptor, beta 1	Synaptic Transmission	QT00007455	4p12b	**−1.77**	<0.0001	0.0086	**−2.85**	0.0003	0.8406	<0.0001
GADD45B	Growth arrest and DNA-damage-inducible, beta	Myeloid differentiation primary response	QT00014084	19p13.3	**−3.64**	0.0001	0.047	**−6.46**	0.024	0.9833	<0.0001
GATA2	GATA binding protein 2	Positive regulation of angiogenesis & phagocytosis	QT00045381	3q21.3	**2.13**	<0.0001	0.0001	**3.49**	0.001	0.5965	0.0314
GJC2	Gap junction protein, gamma 2, 47 kDa	Myelination	QT01674239	1q42.13	**2.25**	0.0001	0.006	**7.38**	0.008	0.9334	<0.0001
HMGCS1	3-hydroxy-3-methylglutaryl-Coenzyme A synthase 1 (soluble)	Metabolic process	QT00055531	5p12	**−1.56**	<0.0001	0.005	**−1.84**	0.019	0.8750	<0.0001
IFNGR1	Interferon gamma receptor 1	Response to virus	QT00089404	6q23.3	**1.55**	<0.0001	0.002	**1.735**	0.015	0.7461	0.0004
JUP	Junction plakoglobin	Cell-cell adhesion	QT00089166	17q21.2	**1.84**	<0.0001	0.00037	**2.95**	0.025	0.82850	0.0016
NOS3	Nitric oxide synthase 3 (endothelial cells)	Angiogenesis	QT00089033	7q36.1-36.1	**1.91**	0.0026	0.0139	**5.05**	0.002	0.8042	0.0028
NOTCH1	Notch homolog 1, translocation-associated (Drosophila)	Notch signalling	QT01005109	9q34.3	**1.62**	<0.0001	0.00029	**1.75**	0.013	0.8656	0.0006
NPTX2	Neuronal pentraxin II	Synaptic transmission	QT00001876	7q22.1	**3.05**	<0.0001	0.002	**3.52**	0.008	0.9275	<0.0001
P2RX7	Purinergic receptor P2X, ligand-gated ion channel, 7	ATP-dependent lysis of macrophages	QT00083643	12q24.31	**2.20**	0.0003	0.0064	**3.12**	0.014	0.9664	<0.0001
PHLDB1	Pleckstrin homology-like domain, family B, member 1	Unknown	QT00083601	11q23.3	**1.52**	0.0013	0.0153	**1.75**	0.023	0.6180	0.0107
PLEKHA5	Pleckstrin homology domain containing, family A member 5	Phosphatidylinositol binding	QT00045605	12p12.3	**−1.57**	<0.0001	0.005	**−2.3**	0.014	0.8098	0.0003
PML	Promyelocytic leukemia	PML body organisation	QT01841945	15q24.1	**1.51**	0.0023	0.0067	**1.69**	0.014	0.8626	0.0006
SEPT3	Septin 3 (neuronal specific)	Unknown	QT00020111	22q13.2	**−1.57**	0.0014	0.01	**−1.87**	0.042	0.8476	<0.0001
SGK1	Serum/glucocorticoid regulated kinase	Response to stress	QT00041293	6q23.2	**1.74**	0.0004	0.0029	**2.07**	0.002	0.587	<0.0001
SNCA	Synuclein, alpha	Dopamine metabolism	QT00035903	4q21	**−1.48**	0.084 ns	ns	**−1.34**	0.35 ns	ns	ns
SNCAIP1	Synuclein, alpha interacting protein	Dopamine metabolism	QT00054320	5q23.2	**−1.62**	0.0012	0.026	**−1.87**	0.055^∧^	0.8388	0.0012
STAT2	Signal transducer and activator of transcription 2, 113 kDa	Response to virus	QT00095704	12q13.2	**1.93**	<0.0001	0.00024	**2.10**	0.011	0.7973	0.0002
TH	Tyrosine hydroxylase	Dopamine biosynthesis	QT00067221	11p15.5	**−3.10**	<0.0001	0.0035	**−3.27**	0.043	0.9016	<0.0001
TJAP1	Tight junction associated protein 1	Tight junction function	QT00091903	6p21.1	**2.03**	<0.0001	0.00079	**1.97**	0.007	0.7145	0.0041
TNFRSF14	Tumour necrosis factor receptor superfamily, member 14 (herpesvirus entry mediator)	Immune response	QT00082432	1p36.32	**1.94**	<0.0001	<0.0001	**2.035**	0.012	ns	ns
ZBTB16	Zinc finger and BTB domain containing 46	Cell cycle progression	QT00029960	11q23.2	**1.67**	0.0011	0.0105	**1.785**	0.032	0.923	<0.0001


^*∧*^ = Trend; ns: not significant

Microarray data: Rosetta error model + Student *t*-test (2-tailed, unequal variance)

qPCR: Student *t*-test (2-tailed, unequal variance)

Correlation microarray and qPCR data: Pearson correlation test.

**Table 4:** Twelve most consistent deregulated genes across microarray studies.

Symbol	Definition	Function	Chromosome	Illumina	Grunblatt et al. 2004 [17]	Hauser et al. 2005 [12]	Zhang et al. 2005 [13]	Moran et al. 2006 (LSN) [14]	Moran et al. 2006 (LSN) [14]	Lesnick et al. 2007 [15]	Cantuti-Castelvetti et al. 2007 [18]	Bossers et al. 2009 [16]
ALDH1A1	Aldehyde dehydrogenase 1 family, member A1	Oxidation reduction	9q21.13	−2.97**	−1.35*		−2.9**	−4.87*	−3.42**	−3.83**	−3.87*	−1.18**
AGTR1	Angiotensin II receptor, type 1	Regulation of vasoconstriction	3q24	−4.15**			−2.1**	−3.57*	−3.7**	−4.34**	−4.34*	−1.09**
ANK1	Ankyrin 1, erythrocytic	Cytoskeleton organisation	8p11.1	−2.23**		−1.35**	−1.58**	−2.34**	−1.95**	−1.6**		
ATP8A2	ATPase, aminophospholipid transporter-like, Class I, type 8A, member 2	ATP biosynthetic process	13q12	−2.06 **		−1.19**	−1.47**	-1.79*	−1.63**			−1.34**
CBLN1	Cerebellin 1 precursor	Synaptic transmission	16q12.1	−2.90**			−1.35**	−2.68**	−1.89**	−2.34*	−2.02*	
ACHE	Acetylcholinesterase (YT blood group)	Synaptic transmission	7q22	−1.82**		−2.08*	−1.15*	−2.72*	−1.96*			
ACOT7	Acyl-CoA thioesterase 7	Lipid metabolism	1p36	−1.79**		−2.28*	−1.26**	−2.6*	−2.04*			
ACSL6	Acyl-CoA synthetase long-chain family member 6	Lipid metabolism	5q31	−2.29**			−1.3*	−1.53**	−1.25**	−1.68*		
ANKZF1	Ankyrin repeat and zinc finger domain containing	Unknown	2q35	1.86**			1.27**	1.38**	1.31**	1.24*		
AP1G2	Adaptor-related protein complex 1	Vesicle-mediated transport	14q11.2	2.11**		1.38*	1.36**		1.18*	1.32**		
APBA3	Amyloid beta (A4) precursor protein-binding, family A, member 3	Synaptic transmission	19p13.3	1.52**			1.22*	1.17*	1.21*	1.21*		
BEX1	Brain expressed, X-linked 1 (BEX1)	Nervous system development	Xq21-q23; Xq22	−1.75**		−1.94*	−1.98**	−2.11**	−1.7*			

*P < 0.05; **P < 0.01.
